# Ablation of the miRNA Cluster 24 Has Profound Effects on Extracellular Matrix Protein Abundance in Cartilage

**DOI:** 10.3390/ijms21114112

**Published:** 2020-06-09

**Authors:** Veronika S. Georgieva, Julia Etich, Björn Bluhm, Mengjie Zhu, Christian Frie, Richard Wilson, Frank Zaucke, John Bateman, Bent Brachvogel

**Affiliations:** 1 Department of Pediatrics and Adolescent Medicine, Experimental Neonatology, Medical Faculty, University of Cologne, 50931 Cologne, Germany; veronika.georgieva@uk-koeln.de (V.S.G.); bluhmbjoern@gmail.com (B.B.); mzhu1@uni-koeln.de (M.Z.); christian.frie@uni-koeln.de (C.F.); 2 Center for Biochemistry, Medical Faculty, University of Cologne, 50931 Cologne, Germany; 3 Dr. Rolf M. Schwiete Research Unit for Osteoarthritis, Orthopaedic University Hospital Friedrichsheim, GmbH, 60528 Frankfurt, Germany; julia.etich@kgu.de (J.E.); frank.zaucke@friedrichsheim.de (F.Z.); 4 Central Science Laboratory, University of Tasmania, Hobart, TAS 7005, Australia; richard.wilson@utas.edu.au; 5 Murdoch Children’s Research Institute, Department of Paediatrics, University of Melbourne, Parkville, VIC 3052, Australia; john.bateman@mcri.edu.au

**Keywords:** extracellular matrix, Mirc24, miR-322, miR-503, cartilage, PRG4, articular, SOX9, SOX6, MMP13

## Abstract

MicroRNAs (miRNAs) regulate cartilage differentiation and contribute to the onset and progression of joint degeneration. These small RNA molecules may affect extracellular matrix organization (ECM) in cartilage, but for only a few miRNAs has this role been defined *in vivo*. Previously, we showed that cartilage-specific genetic ablation of the Mirc24 cluster in mice leads to impaired cartilage development due to increased RAF/MEK/ERK pathway activation. Here, we studied the expression of the cluster in cartilage by LacZ reporter gene assays and determined its role for extracellular matrix homeostasis by proteome and immunoblot analysis. The cluster is expressed in prehypertrophic/hypertrophic chondrocytes of the growth plate and we now show that the cluster is also highly expressed in articular cartilage. Cartilage-specific loss of the cluster leads to increased proteoglycan 4 and matrix metallopeptidase 13 levels and decreased aggrecan and collagen X levels in epiphyseal cartilage. Interestingly, these changes are linked to a decrease in SRY-related HMG box-containing (SOX) transcription factors 6 and 9, which regulate ECM production in chondrocytes. Our data suggests that the Mirc24 cluster is important for ECM homoeostasis and the expression of transcriptional regulators of matrix production in cartilage.

## 1. Introduction

MicroRNAs (miRNAs) are promising targets to control cartilage degeneration and promote its repair. These small single-stranded RNAs can bind to multiple mRNA targets to regulate the activity of entire signalling pathways and cellular responses and may therefore also contribute to cartilage homeostasis. Numerous miRNAs were shown to be associated with osteoarthritis (OA) pathology and computational approaches predict complex interactions with gene signalling networks to mediate cartilage-protective or destructive effects [1,2], but for few miRNAs the biological relevance was demonstrated.

MiR-140 was among the first miRNAs described to be expressed in cartilage and to be downregulated in human OA. The genetic ablation of this miRNA caused a short stature and age-related OA-like phenotype with proteoglycan loss due to elevated ADAMTS5 expression and fibrillation of articular cartilage. The overexpression of miR-140 in cartilage prevented antigen-induced arthritis in transgenic mice [3], further supporting a key role for this miR in cartilage homeostasis. Proteoglycans are highly hydrated macromolecules of the extracellular matrix (ECM) that confer the compression strength to cartilage [4] and miR-140 may protect this network from ADAMTS5-mediated degradation. Therefore, miRNAs can prevent ECM destruction in cartilage in vivo, but may also regulate ECM protein production. Previously, we showed that miR-26a is increasingly expressed in growth plate cartilage to suppress the expression of the ECM adaptor proteins COMP and matrilin-3 in chondrocytes [5], resulting in destabilization of the interaction between the collagen containing fibrillar and the proteoglycan enriched extrafibrillar ECM compartment. We also demonstrated that the cartilage specific genetic ablation of the Mirc24 cluster in mice (Col2a1-Cre-*Mirc24^tm1M^* mice), which encodes for the miRNAs miR-322, miR-351 and miR-503, leads to impaired cartilage development with fatal consequences. The expression of this cluster is upregulated during chondrocyte differentiation and loss of the Mirc24 cluster encoded miR-322 leads to increased RAF/MEK/ERK pathway activation and reduced cartilage growth [6]. The miR-322 was described to target *Igf1r* and *Insr* upstream of the RAF/MEK/ERK pathway in skeletal muscle [7] and vascular smooth muscle cells [8] and to interact with the epigenetic modifier SETD3 to regulate skeletal muscle differentiation [9]. Interestingly, miR-322 stabilizes *Mek1* mRNA to increase MEK1 protein levels and inhibit the activation of the RAF/MEK/ERK pathway in cartilage, but does not regulate IGF1R and INSR levels [6]. Therefore, target specificity of miR-322 may vary between cell types. MiR-351 binds to the mRNA of the vitamin D receptor to inhibit its expression and to modulate osteoblast-mediated bone formation [10] and fibrosis [11], whereas miR-503 targets *Smurf1* to modulate TGF-β signaling and promotes osteogenesis [12]. Hence, Mirc24 cluster encoded miRNAs are important epigenetic factors controlling musculoskeletal development and these miRNAs may also regulate ECM homeostasis in cartilage. [12]

Here, we examined the expression of the Mirc24 cluster in articular cartilage and analyzed the composition of the cartilage ECM in mice where the cluster was conditionally ablated in cartilage (Col2a1-*Mirc24^tm1M^*) to determine its biological relevance for ECM organization.

## 2. Results

### 2.1. The Mirc24 Cluster Is Expressed in Articular Cartilage and Downregulated in Experimental OA

**Figure 1:**
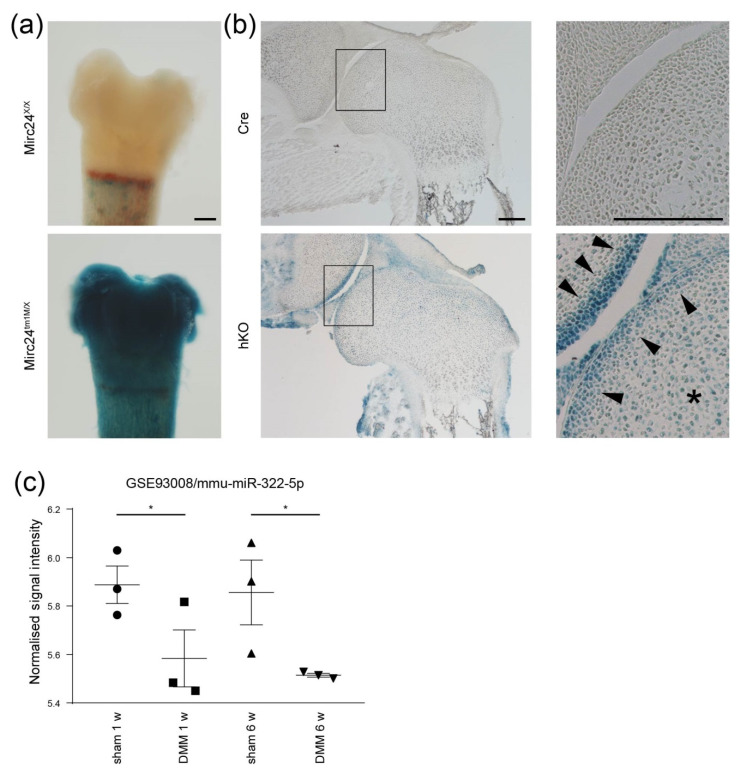
Characterization of the Mirc24 cluster expression in epiphyseal cartilage. Expression of the LacZ reporter gene in (**a**) isolated femora of *Mirc24^X/X^* and *Mirc24^tm1M/X^* female newborns and (**b**) sections of the distal epiphyseal femoral joints of Col2a1-Cre (Cre) and Col2a1-Cre-*Mirc24 ^tm1M/^^Y^* (hKO) newborn males was detected by X-Gal staining and assessed by microscopy. Asterix: epihyseal head cartilage, arrowheads: articular cartilage, bar: (**a**,**b**) 200 µm. (**c**) Expression of the Mirc24 cluster-encoded miR-322-5p in articular cartilage from mice subjected to sham or destabilization of the medial meniscus (DMM) surgery (*n* = 3) at 1 and 6 weeks was determined by Geo2R analysis and expression intensity and *p*-value of the GEO dataset GSE93008 are shown.

To study the expression of the Mirc24 cluster encoded miRNAs in developing cartilage in detail we detected the LacZ reporter gene expression in Mirc24-LacZ^+^ transgenic mice using X-Gal staining on whole mounts of the distal epiphysis of the femur and on sections through joints. The blue X-Gal staining product was detected in the chondrocytes of epiphyseal cartilage (Figure 1a,b asterix) and the staining intensity was increased in the superficial zone of articular cartilage (Figure 1b, arrowheads). Previously, we demonstrated that the cluster is also expressed in differentiating prehypertrophic/hypertrophic chondrocytes [6]. The results indicate that cluster expression is associated with late chondrocyte differentiation in growth plate and articular cartilage.

To detect Mirc24 cluster encoded miRNAs in intact and damaged mature articular cartilage, we analyzed the expression of the cluster-encoded miRNAs miR-322, miR-351 and miR-503 using the expression dataset (GEO DataSet GSE93008) of a mouse model with post-traumatic osteoarthritis (OA) [13]. In this study, expression profiles of miRNAs were determined in medial tibial cartilage from sham or DMM (destabilization of the medial meniscus) operated 10–12-week-old male wild-type C57BL/6 mice. Here, the cluster-encoded miR-322 was detected in the intact cartilage of sham-operated joints at 1 week and 6 weeks and its expression was slightly reduced in cartilage of DMM-operated joints at both time points (Figure 1c). MiR-351 and miR-503 were not detected. Hence, the cluster is expressed also in mature articular cartilage, miR-322 is the predominately expressed miRNA of the cluster and its expression is altered in DMM.

### 2.2. ECM Entities Are Enriched in Proteome Analysis of Mirc24 Cluster-Deficient Epiphyseal Cartilage Extracts

**Figure 2:**
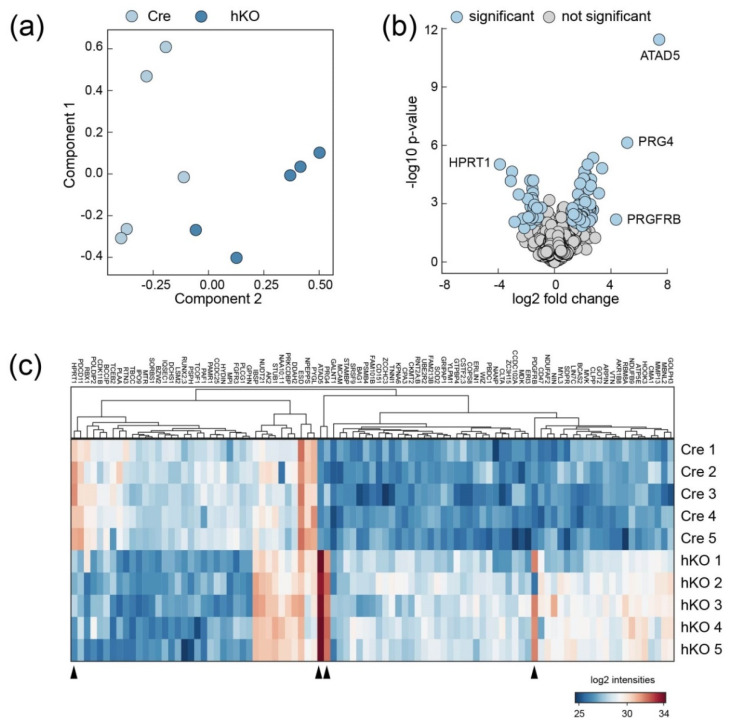
Proteome analysis of epiphyseal cartilage from Mirc24 deficient mice. (**a**) Principal component analysis of the proteome dataset. Each circle represents an individual sample of epiphyseal cartilage isolated from Col2a1-Cre (Cre) or Col2a1-Cre-*Mirc24^tm1M/Y^* (hKO) newborn mice. (**b**) Volcano plot illustrating significant changes within the dataset. Proteins with the highest fold change are marked (blue). (**c**) Non-averaged hierarchical clustered intensity plot (distant metrics—euclidean, linkage rule—complete) of differentially abundant proteins. Protein symbols within the clusters are given and the relative protein abundance is indicated (blue—low, red—high). Proteins with the highest fold change are marked (blue) (arrowheads).

**Figure 3:**
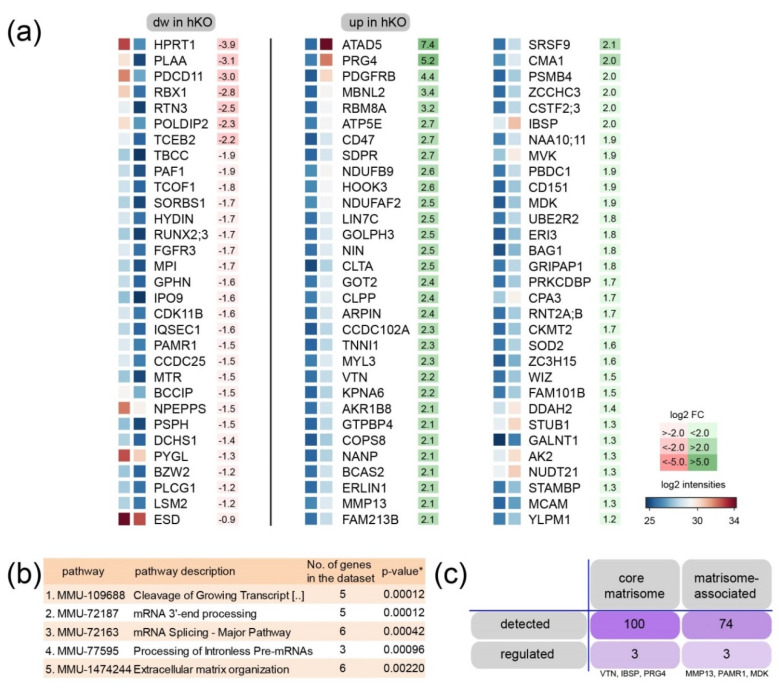
Identification of regulated protein interaction networks in cartilage extracts from Mirc24 deficient mice. (**a**) Proteins with decreased (dw in hKO, left) and increased (up in hKO, right) intensity are ranked according to the fold change. The results of the (**b**) reactome pathway characterization of increased proteins within string database and the (**c**) matrisome analysis of regulated entities are shown.

We hypothesized that the cluster regulates ECM homeostasis in cartilage and therefore studied the ECM composition in mice with cartilage-specific genetic ablation of the Mirc24 cluster. To define changes in protein abundance we first isolated femoral and tibial epiphyses of Col2a1-Cre (Cre) and Col2a1-Cre-*Mirc24^tm1M/Y^* (hemizygous, hKO) newborns and subjected the samples to proteome analysis using established protocols [14]. Samples of five biological replicates were deglycosylated followed by extraction with 4 M GuHCl to isolate ECM molecules from epiphyses. After trypsin digest samples were assayed by mass spectrometry and the generated data set was analyzed using MaxQuant and Perseus software. Principal component analysis shows that data sets were substantially different between genotypes. Extracts from Col2a1-Cre-*Mirc24^tm1M/Y^* cartilage separate well from extracts from Col2a1-Cre cartilage (Figure 2a). In total 2260 proteins were identified in epiphyseal cartilage and among those 93 were differentially abundant between femoral epiphyseal cartilage extracts from Col2a1-Cre and Col2a1-Cre-*Mirc24^tm1M/Y^* mice, respectively (Q < 0.05 sO 0.2) (Figure 2b, Supplemental Table S1)). Hierarchical cluster analysis determined a cluster of 31 proteins with decreased abundance and a cluster of 62 proteins with increased abundance in cartilage from Col2a1-Cre-*Mirc24^tm1M/Y^* mice (Figure 2c). Most proteins showed changes of ~2-fold in protein abundance with several others that were more strongly decreased or increased. Interestingly, among the more highly regulated proteins the ECM proteoglycan 4 (PRG4—synonyms lubricin, superficial zone protein) showed a 5-fold increase in cartilage extracts of Col2a1-Cre-*Mirc24^tm1M/Y^* mice (Figure 3a). Next, we used string-implemented reactome pathway enrichment analysis [15,16]. While no enriched pathways were detected for the cluster with decreased protein abundance, 10 enriched pathways were found for the cluster with increased abundance. Pathways involved in mRNA-cleavage (1.), -processing and –splicing (2.–4.) were significant and extracellular matrix organization (5.) was among the highest ranked pathways (Figure 3b). Based on this, a full analysis of the detected matrisome [17,18] was used to determine ECM constituents with changed abundance in cartilage extracts of Col2a1-Cre-*Mirc24^tm1M/Y^* mice. In total six matrisome proteins were identified as altered, including three proteins of the core matrisome (PRG4, bone sialoprotein 2 (IBSP), vitronectin (VTN) and three proteins of the matrisome-associated data set (matrix metallopeptidase 13 (MMP13), peptidase domain containing associated with muscle regeneration 1 (PAMR1) and midkine (MDK) (Figure 3c).

### 2.3. ECM Composition and SOX 6/9 Expression Is Altered in Mirc24-Cluster Deficient Epiphyseal Cartilage

**Figure 4:**
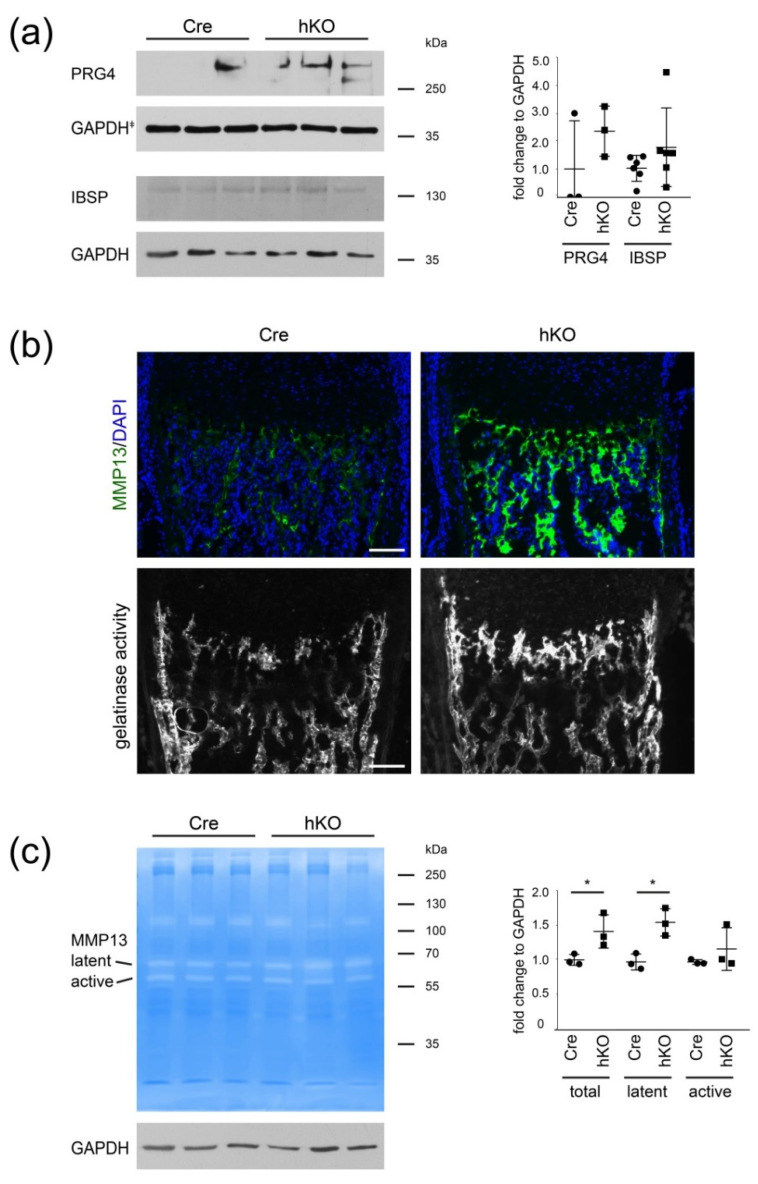
Characterization of PRG4, IBSP and MMP13 levels in femoral joint cartilage. (**a**) PRG4 and IBSP protein levels were determined in newborn cartilage extracts from Col2a1-Cre (Cre) and Col2a1-Cre-*Mirc24^tm1M^^/Y^* (hKO) mice using immunoblots. GAPDH was used as loading control. Quantification is shown (graph, PRG4 *n* = 3, IBSP *n* = 6). GAPDH^‡^ indicates that PRG4, SOX6 and SOX9 were tested on a single blot and the same GAPDH blot is shown in Figure 4a and Figure 5c. Additional immunoblots are shown in Supplemental Figure S3a. (**b**) MMP13 localization was studied by immunofluorescence analysis (DAPI—blue, MMP13—green, pseudocolored) and gelatinase activity was determined by in situ zymography in newborn femoral epiphysis. Brightness was adjusted for visualization. (**c**) MMP13 activity in femoral extracts was analyzed by gelatin zymography. Latent proMMP13 and active MMP13 are indicated. GAPDH was used as loading control. Fold change of total, latent and active MMP13 in hKO extracts compared to Cre was determined (graph, *n* = 3). Molecular weights of Thermo Scientific™ PageRuler™ Plus Prestained 10–250 kDa Protein Ladder bands are given. Scale bar 100 µm. *p* < 0.05 (*).

To validate the results of the proteome analysis cartilage extracts were also analyzed by immunoblotting using antibodies against PRG4 and IBSP. PRG4 is a highly glycosylated protein with different isoforms [19] and we could detect increased levels of presumably monomeric proteins at an apparent molecular weight above 250 kDa in Mirc24 mice (Figure 4a). No changes in IBSP protein levels in cartilage extracts of Col2a1-Cre-*Mirc24^tm1M/Y^* mice were detected when compared with extracts of Col2a1-Cre mice. MMP13 expression was studied by immunofluorescence analysis using sections of the femoral growth plate of Col2Cre and Col2a1-Cre-*Mirc24 ^tm1M/Y^* mice. Here a significant increase in signal intensity in late hypertrophic growth plate cartilage and the bone marrow of Col2a1-Cre-*Mirc24 ^tm1M/Y^* mice was observed (Figure 4b and Supplemental Figure S2a). In situ zymography indicated that gelatinase activity was increased at these sites of MMP13 expression in cartilage and bone (Figure 4b and Supplemental Figure S2b) and in-gel zymography showed for the latent and active MMP13 that clear bands against a blue background of undigested substrate were detected in femoral extracts of Col2Cre and Col2a1-Cre-*Mirc24 ^tm1M/Y^* mice. The activity of the latent form was increased in Col2a1-Cre-*Mirc24 ^tm1M/Y^* newborn mice compared to control (Figure 4c, quantification).

**Figure 5:**
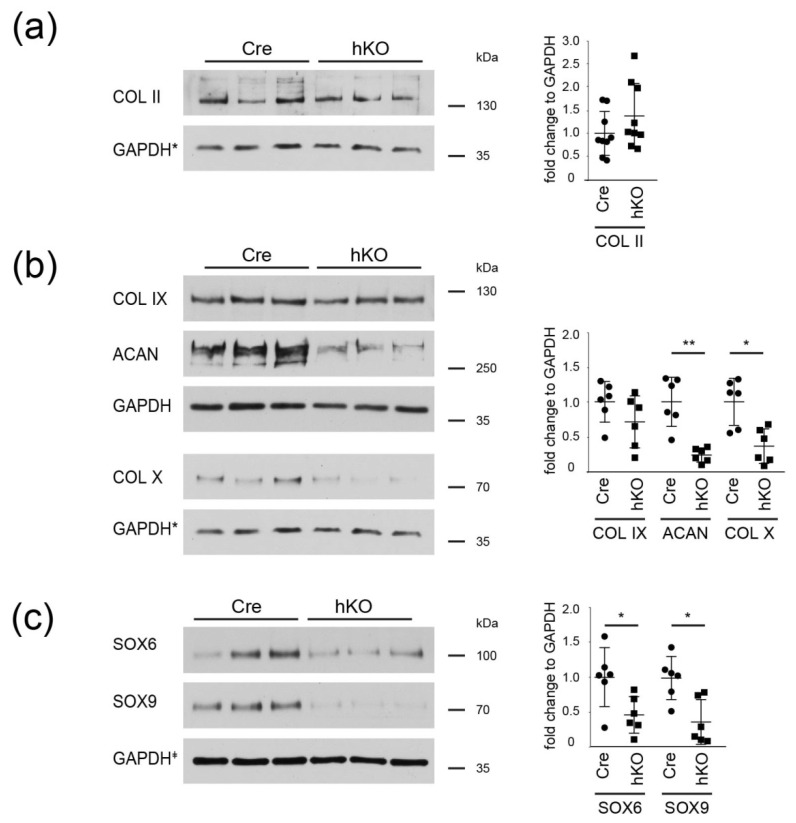
Analysis of the ECM and SOX protein expression in cartilage. Cartilage extracts from Col2a1-Cre (Cre) and Col2a1-Cre-*Mirc24^tm1M/Y^* (hKO) mice were analyzed for the presence of (**a**) collagen II, (**b**) collagen IX, aggrecan, and X as well as (**c**) SOX6 and SOX9 using immunoblots. GAPDH was used as loading control. GAPDH* indicates that Collagen II and X were tested on a single blot and the same GAPDH blot is shown in (**a**) and (**b**). GAPDH^‡^ indicates that PRG4, SOX6 and SOX9 were tested on a single blot and the same GAPDH blot is shown in Figure 4a and Figure 5c. Quantification is shown (graph, *n* ≥ 6). Additional immunoblots are shown in Supplemental Figure S3b–d. Molecular weights of Thermo Scientific™ PageRuler™ Plus Prestained 10–250 kDa Protein Ladder bands are given. *p* < 0.05 (*), *p* < 0.01 (**).

To further expand the characterization of the extracellular matrix and identify changes that were maybe not detected by proteome analysis, the abundance of fibrillar and extrafibrillar ECM were characterized in immunoblots (Figure 5 and Supplemental Figure S3). The abundance of the fibrillar collagen II and the adaptor protein collagen IX was not significantly altered, whereas levels of the extrafibrillar proteoglycan aggrecan and the network forming collagen X were significantly reduced in cartilage extracts from Col2a1-Cre-*Mirc24^tm1M^* mice. The results show that these components of the cartilage ECM are decreased in cartilage extracts of Col2a1-Cre-*Mirc24 ^tm1M/Y^* mice. ECM production in cartilage is controlled by SRY-related HMG box-containing (SOX) transcription factors and we also determined protein levels of the key transcription factor regulating ECM production, SOX9, and its cofactor SOX6 using immunoblots. Total levels of both transcription factors were strongly decreased in cartilage extracts of Col2a1-Cre-*Mirc24^tm1M/Y^* mice. Hence, loss of the cluster Mirc24 cluster in cartilage reduces the expression of key transcriptional regulators in ECM formation.

## 3. Discussion

Despite the extensive knowledge of genetic factors controlling ECM/cartilage formation, maintenance and repair, the role of epigenetic factors in vivo has yet to be determined. Here, we studied the consequences of the Mirc24 cluster deficiency for ECM homeostasis in cartilage and show that this cluster is important for ECM organization.

Previously, we identified expression of the Mirc24 cluster in prehypertrophic/hypertrophic growth plate cartilage [6] and we now demonstrate that the cluster is also highly expressed in articular cartilage. Hence, cluster expression is closely linked to chondrocyte differentiation. The cluster encodes several miRNAs, but among the cluster-encoded miRNAs only miR-322 and miR-503 are expressed in prehypertrophic/hypertrophic growth plate cartilage and only miR-322 is expressed in mature articular cartilage in 10–12 week old male WT C57BL/6. Here, aging and genetic background may contribute to the differential expression of cluster-encoded miRNAs. The observed effects on ECM organization in cartilage of Mirc24 deficient mice could be attributed to the loss of the two cluster-encoded miRNAs miR-322 and miR-503 in epiphyseal cartilage. Furthermore, the reduced levels of miR-322 in the lesional cartilage from mice where OA is induced by meniscal destabilization, and correspondingly reduced miR-322 and miR-503 in the OA subchondral bone [13] may implicate these components of the Mirc24 cluster in cartilage homeostasis and degeneration.

We hypothesized that cluster-encoded miRNAs are likely to regulate ECM production in differentiated chondrocytes and this was supported by reactome pathway analysis of the proteome dataset. Interestingly, of the components belonging to the significant pathway “ECM organization” that were altered in Col2a1-Cre-*Mirc24 ^tm1M/Y^* mice, none are predicted targets of the cluster-encoded miRNAs [20]. In addition, the majority of the cluster-encoded miRNAs inhibit expression of their respective target genes. Therefore, the observed increase in these ECM components is presumably secondary to the changes in target gene expression. The proteoglycan PRG4 showed the strongest increase (Figure 3a, ~5-fold) among the ECM proteins in cartilage extracts from Col2a1-Cre-*Mirc24 ^tm1M/Y^* mice. This protein is extensively glycosylated with attachment sites for chondroitin and keratan sulfate chains and with the capability to from multimers [19]. We confirmed by immunoblot an increase in the levels of monomeric species of the appropriate molecular size but this was not accompanied by changes in Prg4 mRNA expression (see Supplemental Figure S1) in chondrocytes of Col2a1-Cre-*Mirc24 ^tm1M/Y^* mice. Therefore, we hypothesize that cluster encoded miRNAs affect post-translational PRG4 modification that might alter protein stability or deposition within the articular cartilage matrix.

The cluster may also be important for ECM degradation. MMP13 protein levels were significantly increased in proteome analysis and by immunofluorescence we observed an increased MMP13 signal intensity at the chondroosseus border and the bone marrow of Col2a1-Cre-*Mirc24 ^tm1M/Y^* mice compared to control, as well as increased gelatinase activity at the sites of MMP13 expression. Several cartilage ECM substrates are degraded by MMP13 and among those collagen II, aggrecan and collagen X are targeted by MMP13 to remodel the ECM and promote cartilage to bone transformation [21,22]. We observed a reduction of collagen X and aggrecan by immunoblot and this could be linked to increased MMP13-mediated degradation of these ECM molecules at the chondroosseus junction of Col2a1-Cre-*Mirc24 ^tm1M/Y^* mice. The decrease in collagen X is also in line with our previous results, where a significant decrease in collagen X-positive hypertrophic zone was seen in immunostainings of the chondroosseous junction of Col2a1-Cre-*Mirc24 ^tm1M/Y^* mice [6]. Conversely, no decrease in collagen II levels was detected, but the mature collagen fibrils of the cartilage ECM may be less susceptible to early cleavage events due to the presence of numerous adaptor proteins and other small proteoglycans. While collagen II protein levels were similar between Col2a1-Cre-*Mirc24 ^tm1M/Y^* mice compared to control, the transcription factors SOX9 and SOX6 were reduced. These changes were not apparent in the proteome analysis but differences in detection may be due to technical differences in sample treatment and sensitivity between immunoblot and proteome analysis [23].

We observed a decrease of SOX6 and SOX9 protein levels in epiphyseal cartilage of Col2a1-Cre-*Mirc24 ^tm1M/Y^* mice by immunoblot and these proteins are essential transcriptional regulators of cartilage differentiation and ECM production. SOX9 is required for chondrocyte proliferation and hypertrophy and the decrease in SOX9 levels may contribute to the significant decrease in the total area of hypertrophic cells in femora of Col2a1-Cre-*Mirc24 ^tm1M/Y^* mice [6]. SOX9 also regulates the expression of its own partners in transcriptional regulation, SOX6 and SOX5 [24] and the SOX trio act in concert to transactivate cartilage matrix genes including those encoding aggrecan [25] and collagen X [26]. Hence, the decrease in SOX9 and SOX6 may directly inhibit ECM production and therefore affect the stability of the cartilage ECM in Col2a1-Cre-*Mirc24 ^tm1M/Y^* mice.

miRNAs are often seen as potential therapeutic opportunity for OA and other cartilage degenerative diseases, but regulation by miRNAs is complex and may include cartilage-protective and destructive mechanisms and signaling pathways [1] adding another level of complexity to the posttranscriptional regulation of cartilage development and homeostasis. In this study we show that miRNAs of the Mirc24 cluster, in particular miR322, which is dysregulated in mouse OA cartilage, has a potentially important role in cartilage ECM protective and destructive processes in vivo.

## 4. Materials and Methods

### 4.1. Mice

MicroRNA cluster 24 transgenic mice (*Mirc24^tm1M/tm1M^*), containing a LacZ reporter gene and lox sites to inactivate the cluster-encoded miR-322, miR-351 and miR-503 [27] and Col2a1-Cre mice [28] were used for animal studies. Breeding protocols to generate hemizygous males Col2a1-Cre-*Mirc24^tm1M/Y^* and Col2a1-Cre littermates were previously described [6]. All animal experiments were performed in accordance with the animal ethics guidelines of the German animal protection law. Institutional review board - Landesamt für Natur, Umwelt und Verbraucherschutz Nordrhein-Westfalen.

### 4.2. In Situ Detection of LacZ Reporter Gene Activity

Isolated femora from newborn mice were fixed for 15 min in 0.2% glutaraldehyde, 5 mM EGTA, 2 mM MgCl_2_, 0.1 M phosphate buffer, pH 7.3, washed three times with 2 mM MgCl_2_, 0.02% NP-40, 0.1 M phosphate buffer, pH 7.3 and stained in 50 mg/mL X-gal, 1 mM potassium ferricyanide, 2 mM MgCl_2_, 0.02% NP-40, 0.1 M phosphate buffer, pH 7.3 overnight at room temperature. Whole mount stainings were assessed by microscopy. In addition, isolated femora were embedded in O.C.T. Tissue-Tek (Sakura Finetek, Tokyo, Japan), sectioned using a Leica Cryotome CM3050. Sections (7 µm) were incubated for 15 min in fixative and then stained for 16 h at RT and 10 h at 37 °C in X-Gal staining solution. Sections were washed in 70% (*v*/*v*) ethanol and dH_2_O, mounted in Mowiol^®®^ 4-88 (Sigma-Aldrich, St. Louis, MI, USA) and analyzed by microscopy (Nikon Eclipse TE2000-U Microscope, Nikon, Tokyo, Japan).

### 4.3. miRNA Expression Analysis in the GEO Dataset GSE93008

The GEO dataset GSE93008 was originally generated by inducing osteoarthritis in adult male C57BL6 mice using the destabilization of the medial meniscus (DMM) [13]. RNA from medial tibial cartilage after sham or DMM surgery was isolated by dissection at 1 and 6 weeks after injury (*n* = 3/time point/group) and the expression was studied using Agilent microarrays [13]. GEO2R analysis tool (https://www.ncbi.nlm.nih.gov/geo/geo2r) was used to compare sham and DMM samples and to identify differentially expressed miRNAs of the Mirc24 cluster.

### 4.4. Protein Extraction

Femoral and tibia knee cartilage from newborns was dissected and stored at −80 °C. Protein extraction was performed as previously described [14]. Briefly, epiphyseal cartilage was lysed using TissueLyser II (QIAGEN, Hilden, Germany) and incubated with 100 µL 100 mM tris-acetate buffer, pH 8.0, 10 mM EDTA, 0.1 U Chondroitinase ABC (Sigma-Aldrich, St. Louis, Missouri, USA) and deglycosylated for 6 h at 37 °C. Protein extraction was performed with 500 µL chaotropic guanidine buffer (4 M GuHCl, 65 mM DTT, 10 mM EDTA, 50 mM sodium acetate, pH 5.8) for 24 h at 4 °C. Protein extracts were precipitated in 9 volumes of ice cold 96% (*v*/*v*) ethanol overnight at −20 °C, washed twice with 70% (*v*/*v*) ethanol and resuspended in 8 M Urea, 50 mM triethyl ammonium bicarbonate for proteome analysis or in 7 M urea, 2 M thiourea, 4% CHAPS, 30 mM Tris, pH 8.0 for immunoblot analysis.

### 4.5. Proteome Analysis

Approximately 15 μg of protein/biological sample were sequentially reduced, alkylated, and digested in Lys-C and trypsin using standard procedures. Peptide samples (*n* = 5 biological replicates per genotype) were analyzed by liquid chromatography-MS on a Q Exactive Plus Orbitrap (Thermo Fisher Scientific, Waltham, MA, USA) mass spectrometer coupled with an EASY nLC 1000 (Thermo Fisher Scientific, Waltham, MA, USA). The Orbitrap was operated in data-dependent acquisition mode. Peptides were loaded onto an analytical column (self-packed, 50 cm–75 µm I.D., filled with 2.7 µm Poroshell EC120 C18, Agilent) using 0.1% formic acid as loading buffer. Peptides were chromatographically separated with a gradient 3–5% 0.1% formic acid in 80% acetonitrile over 1 min and a constant flow rate of 250 nL/min, followed by 5–30% over 65 min, 30–50% over 13 min, 50–95% over 1 min, washing and column equilibration. The MS1 survey scan was acquired with a resolving power set to 70,000 in the mass range from *m*/*z* 300 to *m*/*z* 1750. The top 10 most abundant peptides were isolated within a 1.8 Th window and subjected to HCD fragmentation at normalized collision energy of 27%. The AGC target was set to 5e5 charges, allowing a maximum injection time of 55 ms. Product ions were detected in the Orbitrap at a resolution of 17,500. Precursors were dynamically excluded for 15.0 s. Peptides were identified by searching expected protein sequences in the UniProt Mouse database using MaxQuant software (version 1.5.3.8, https://www.maxquant.org/). Standard settings were used (in short, 1% FDR using a reversed database with razor approach, LFQ quantification and normalization using MaxLFQ algorithm [29] with activated match between runs option between samples from the Cre control and hKO groups, respectively.

Afterwards, results from the protein group table were processed in Perseus (version 1.6.1.1, https://www.maxquant.org/perseus). After initial basic data cleanup (removal of identifications stemming from peptides only identified in modified form, or identified as contaminants, or from the reverse database used for FDR calculations), all LFQ intensities were log2 transformed and samples grouped together either in the Cre control or hKO group. All proteins not present in at least four out of five replicates in at least one group were filtered out. Each sample was checked for normal distribution of intensities before and after imputation of missing values, which was performed for each sample individually. A two-sample Student’s *t*-test was performed with a permutation-based FDR of 0.05, 250 randomizations, and an S0 of 0.2. Proteins with a resulting q-value below 0.05 (93 in total) were deemed significantly different between the two groups. The data were imported in InstantClue (version 0.5.3, [30]) for visualization by principal component analysis, volcano plot and hierarchical clustering. For volcano plot –log10 *p*-values were plotted against the log2 fold change (a q-value < 0.05 was considered significant). Hierarchical cluster analysis was performed with the log2 transformed LFQ intensities using the following settings: distant metrics—euclidean, linkage rule—complete. Reactome analysis of significantly regulated proteins was performed using the String database (http://string-db.or). Matrisome gene clusters, annotated in the matrisome database 2.0 (http://matrisomeproject.mit.edu) were imported in FunRich (version 3.1) to generate graphical representations of altered core matrisome and matrisome associated proteins. The mass spectrometry proteomics data have been deposited to the ProteomeXchange Consortium via the PRIDE [31] partner repository with the dataset identifier PXD019374.

### 4.6. RNA Isolation and Semiquantitative PCR

Total RNA from frozen femoral and tibial epiphyseal cartilage of each animal was extracted by phenol-chloroform extraction using TRIzol reagent (Thermo Fisher Scientific, Waltham, MA, USA). The concentration of RNA was determined using NanoDrop 2000 spectrophotometer (Thermo Fischer Scientific, Waltham, MA, USA). RNA was reversely transcribed into cDNA (Omniscript RT assay, QIAGEN, Hilden, Germany) and 25 ng cDNA was used for REDTaq^®®^ ReadyMix™ PCR assays (Sigma-Aldrich, St. Louis, MI, USA). The expression was normalized to ß-actin (*Actb*). The following primers were used: *Actb* (forward: 5′-GACGAGGCCCAGAGCAAGAG-3′; reverse: 5′-CTAGAGCAACATAGCACAGC-3′) and *Prg4* (forward: 5′-GTGGATGAAGCTGGAAGCGG-3′; reverse: 5′-GTTGGAGGTGGTTCCTTGGG-3′.

### 4.7. Fluorescent In Situ Hybridization Analysis

Detection of *Prg4* transcripts on cryotome sections (7 µm) was conducted using QuantiGene^®®^ ViewRNA ISH Tissue Assay Kit and QuantiGene^®®^ ViewRNA ISH Cell Assay Kit (Affymetrix, Santa Clara, CA, USA) according to the instruction of the manufacturer. An Alexa Fluor 488 (Affymetrix, USA) labeled probe targeting nucleotides 2721–3891 of the murine transcript variant 1 (NM_021400.3,) was used to detect PRG4 expression in situ. Stained sections were analyzed by immunofluorescence microscopy (Nikon Eclipse TE2000-U Microscope, Nikon, Tokyo, Japan).

### 4.8. Immunoblot Analysis

Cartilage extracts (*n* ≥ 3 biological replicates per genotype) were incubated in SDS sample buffer (62.5 mM Tris buffer, pH 6.8, 2% SDS, 10% glycerol, 0.04% bromophenol blue, 0.5% β-mercaptoethanol, 50 mM DTT) for 15 min at 65 °C, resolved on 8% SDS polyacrylamide gels and transferred onto nitrocellulose (Whatman, Sigma-Aldrich, St. Louis, MI, USA). Primary antibodies against PRG4 (1/150, Invitrogen, Carlsbad, CA, USA), IBSP (1/500, Immundiagnostik, Bensheim, Germany), SOX6 (A4, 1/1000, Santa Cruz Biotechnology, Dallas, TX, USA), SOX9 (1/1000, Millipore, Burlington, MA, USA), aggrecan (1/1000, Millipore, Burlington, Massachusetts, USA), collagen IX (1/2000, [32]), collagen II (1/100, Abcam, Cambridge, UK), collagen X (X53, 1/500, [33]) and GAPDH (14C10, 1/1000, Cell Signaling Technology, Danvers, MA, USA) were added overnight at 4 °C. Primary antibodies were detected by corresponding secondary antibodies labeled with horseradish peroxidase (DAKO, Jena, Germany) and visualized by enhanced chemoluminescence using 10 mM Tris, pH 8.8, 12.5 μM luminol, 2.3 μM coumarin acid, 5.3 μM hydrogen peroxide solution. Band intensities were quantified by ImageJ software [34]. A density profile line graph was obtained and the area under each peak was measured as the number of pixels, normalized to GAPDH and calibrated by fixed point of control [35].

### 4.9. Gelatin Zymography

Femora extracts (*n* = 3 biological replicates per genotype) were lysed in 50 mM Tris buffer, pH 7.5, 150 mM NaCl, 1% (*w*/*v*) SDS, 0.5% (*v*/*v*) NP-40, mixed with non-reducing SDS sample buffer (62.5 mM Tris buffer, pH 6.8, 2% (*w*/*v*) SDS, 10% (*w*/*v*) glycerol, 0.04% bromophenol blue) for 10 min at RT and resolved on 8% SDS polyacrylamide gels copolymerized with 1 mg/mL gelatin (bovine skin type B, Sigma-Aldrich, St. Louis, MI, USA) as previously described [36]. Gels were washed three times in 2.5% (*v*/*v*) Triton X-100 for 20 min at RT to remove residual SDS and renaturate MMPs and then incubated in 50 mM Tris buffer, pH 7.4, 10 mM CaCl_2_, 0.02% (*w*/*v*) NaN_3_ for 3 h at 37 °C. Gels were stained with Coomassie Blue R-250 and latent proMMP13 and active MMP13 gelatinase activity was detected as clear bands against a blue background. The image was converted to 8 bit and band intensities were quantified by ImageJ software [34].

### 4.10. In Situ Zymography

Femora from newborn mice (*n* = 4 biological replicates per genotype) were isolated, embedded in O.C.T. Tissue-Tek (Sakura Finetek, Tokyo, Japan) and sectioned using a Leica Cryotome CM3050. Sections (7 µm) were washed three times in PBS and incubated with 20 µg/mL DQ gelatin (D-12054, Invitrogen) in 50 mM Tris buffer, pH 7.6, 150 mM NaCl, 5 mM CaCl_2_, and 0.2 mM NaN_3_ overnight at 37 °C. To verify the contribution of matrix metallopeptidases, control slides were incubated with 20 mM EDTA for 30 min at RT prior to addition of substrate containing 20 mM EDTA. Slides were incubated for 10 min in the dark at RT in 4% paraformaldehyde, stained with DAPI, embedded in mounting medium and analyzed by immunofluorescence microscopy (Nikon Eclipse TE2000-U Microscope, Nikon, Tokyo, Japan).

### 4.11. Immunofluorescence Analysis

Femora from newborn mice (*n* = 4 biological replicates per genotype) were isolated, fixed in 4% paraformaldehyde overnight, embedded in paraffin and sectioned using a microtome (HM355 S, ThermoFisher Scientific). Deparaffinized sections (7 μm) were digested with hyaluronidase (5 mg/mL) for 30 min at 37 °C (Sigma-Aldrich, St. Louis, MI, USA), permeabilized with 0.25% TritonX-100 in TBS for 10 min and incubated with 10% FCS and 5% normal goat serum blocking solution. Sections were incubated with a primary MMP13-specific antibody (1/500, Abcam, Cambridge, UK) and the corresponding secondary antibody coupled to Cy3 (Jackson ImmunoResearch, West Grove, PA, USA) using standard protocols. Sections were embedded in mounting medium and analyzed by immunofluorescence microscopy (Nikon Eclipse TE2000-U Microscope, Nikon, Tokyo, Japan).

### 4.12. Statistical Analysis

The Mann-Whitney-U-test was used to test for significance between two groups of samples (IBM SPSS Statistics for Windows, version 26.0; Armonk, NY, USA: IBM Corp.). *p*-values of *p* < 0.05~(*) and *p* < 0.01~(**) were considered as statistically significant. Mean values and standard deviations are given (GraphPad Prism version 7.00 for Windows, GraphPad Software, La Jolla, CA, USA, www.graphpad.com).

## Supplementary Materials

The following are available online at https://www.mdpi.com/1422-0067/21/11/4112/s1.

## Abbreviations

ACAN	Aggrecan
ADAM	A disintegrin and metalloproteinase
ADAMTS	ADAM with thrombospondin motifs
COL	Collagen
DMM	Destabilization of the medial meniscus
DTT	Dithiothreitol
ECM	Extracellular matrix
EDTA	Ethylenediaminetetraacetic acid
EGTA	Ethylene glycol-bis(β-aminoethyl ether)-N,N,N′,N′-tetraacetic acid
FC	Fold change
FCS	Fetal calf serum
GAPDH	Glyceraldehyde 3-phosphate dehydrogenase
GuHCl	Guanidinium hydrochloride
hKO	Hemizygous knock-out
IBSP	Bone sialoprotein 2
MDK	Midkine
MgCl_2_	Magnesium chloride
miR, miRNA	MicroRNA
MMP	Matrix metallopeptidase
MS	Mass spectrometry
OA	Osteoarthritis
SDS	Sodium dodecyl sulfate
SOX	SRY-related HMG box-containing
TBS	Tris-buffered saline
VTN	Vitronectin
