# Supplementary material for: Ablation of the miRNA Cluster 24 Has Profound Effects on Extracellular Matrix Protein Abundance in Cartilage
Source: Int J Mol Sci. 2020 Jun 9;21(11):4112. doi: 10.3390/ijms21114112 (PMC7312048; doi:10.3390/ijms21114112)
Supplement: Supplementary file 1 [file ijms-21-04112-s001.zip › Supplementary File/ijms-762046-SI-final.pdf]

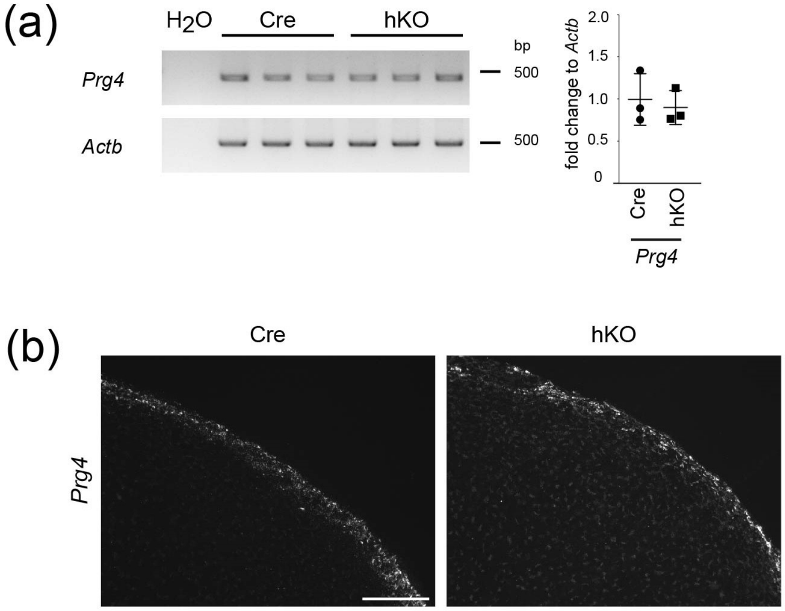

Supplemental Figure 1. *Prg4* mRNA expression in femoral cartilage. (a) Semiquantitative PCR analysis using RNA isolated from newborn cartilage from Col2a1-Cre (Cre) and Col2a1-Cre-Mirc24$^{tm1M/Y}$ (hKO) mice. **(b)** Fluorescence *in situ* hybridization of the femoral articular surface using *Prg4* specific fluorescence probe. Bar: 100 μm.

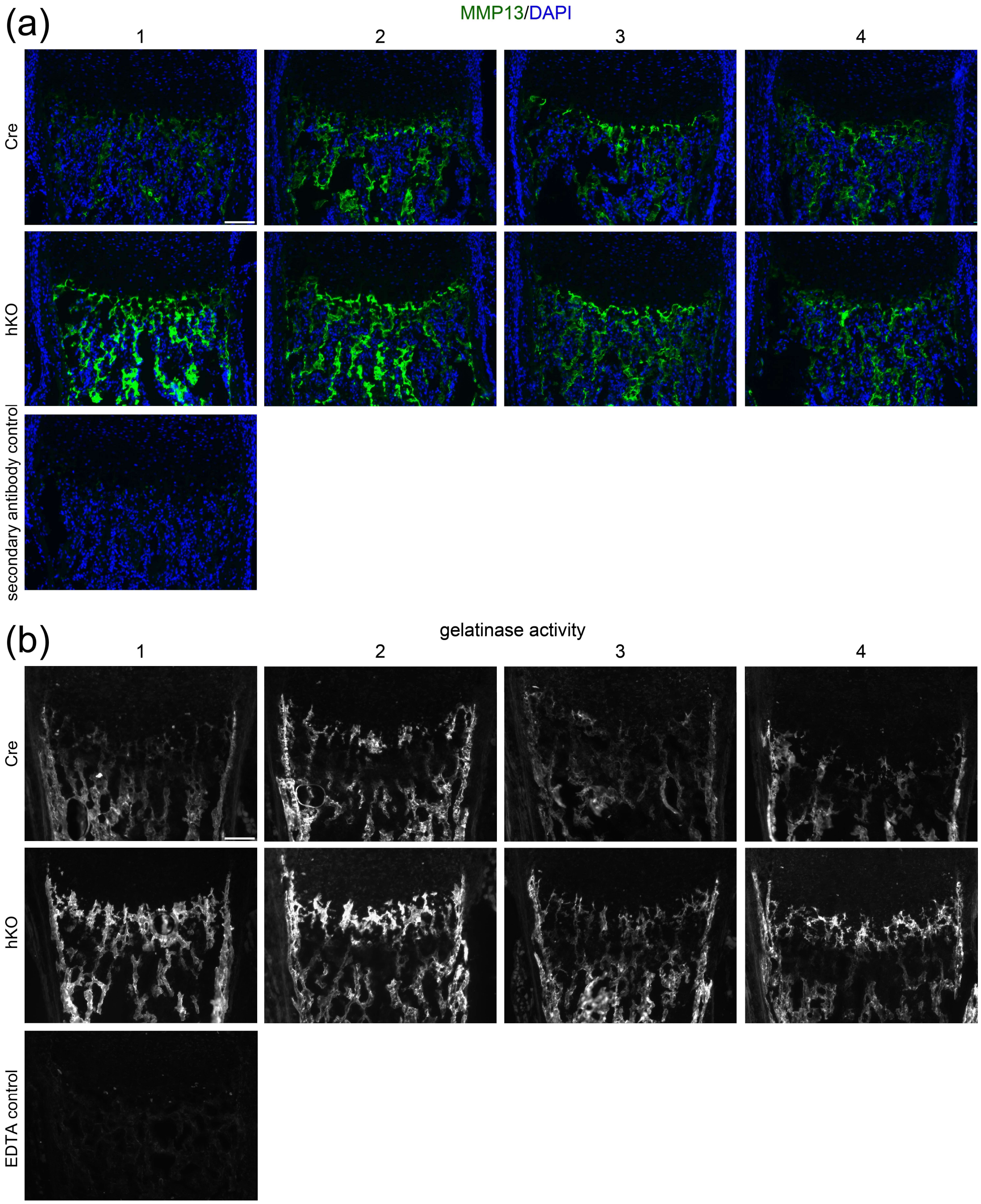

Supplemental Figure 2. Characterization of MMP13 distribution and activity in newborn femora. **(a)** MMP13 localization was studied by immunofluorescence analysis (DAPI - blue, MMP13 - green, pseudocolored) on sections of newborn femora of Col2a1-Cre (Cre) and Col2a1-Cre-Mirc24$^{tm1M/Y}$ (hKO) mice (n=4 per genotype). Secondary antibody control is shown. Brightness was adjusted for visualization. (**b**) Gelatinase activity was determined by *in situ* zymography on sections of newborn femora of Col2a1-Cre (Cre) and Col2a1-Cre-Mirc24$^{tm1M/Y}$ (hKO) mice (n=4 per genotype). Control slide treated with EDTA is shown. Scale bar 100 μm.

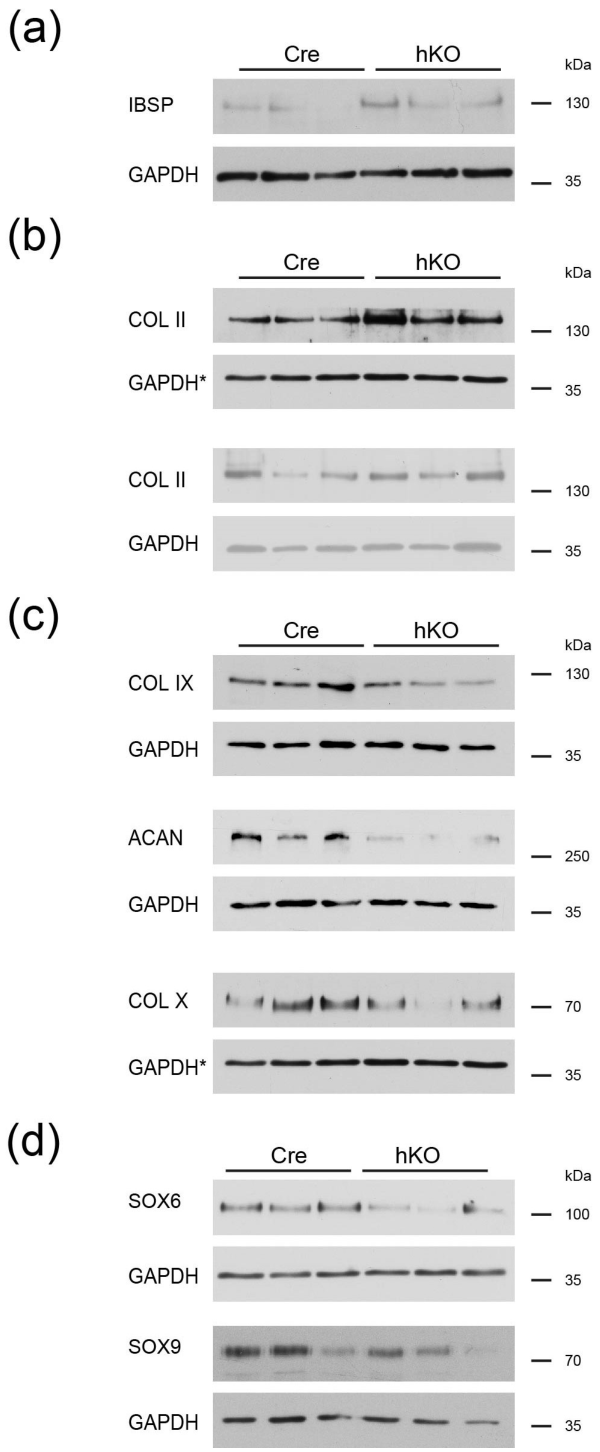

Supplemental Figure 3. Immunoblots for the quantification of IBSP, ECM and SOX protein abundance in cartilage in Fig. 4 and Fig. 5. Additional cartilage extracts from Col2a1-Cre (Cre) and Col2a1-Cre-Mirc24$^{tm1M/Y}$ (hKO) mice were analyzed for the presence of **(a)** IBSP (**b**) collagen II, **(c)** collagen IX, aggrecan, and collagen X as well as **(d)** SOX6 and SOX9 using immunoblots. GAPDH was used as loading control. Molecular weights of Thermo Scientific™ PageRuler™ Plus Prestained 10-250 kDa Protein Ladder bands are given.
